# Changes in Active Components, Antioxidant Activity and Alcohol Dehydrogenase Activity of *Penthorum chinense* Pursh at Different Harvest Times

**DOI:** 10.3390/foods15081371

**Published:** 2026-04-15

**Authors:** Zhuoya Xiang, Hongchun Liang, Qian Lai, Junlin Deng, Lu Gan, Yongqing Zhu, Yinghao Yuan, Chen Xia, Manyou Yu

**Affiliations:** 1 Institute of Agro-Products Processing Science and Technology (Institute of Food Nutrition and Health), Sichuan Academy of Agricultural Sciences, 60 Shizishan Road, Chengdu 610066, China; 2 Agricultural and Rural Affairs Bureau of Gulin County, Gulin 646500, China

**Keywords:** *Penthorum chinense*, harvesting period, active components, antioxidant activity, alcohol dehydrogenase activity

## Abstract

This study aims to establish a time-resolved harvesting standard for *Penthorum chinense*. To achieve this, we systematically integrated growth phenology, phytochemical accumulation dynamics, and antioxidant activity across six key developmental stages. The contents of total phenolics, flavonoids, proanthocyanidins, and tannins exhibited a biphasic fluctuation pattern, which was closely correlated with variations in antioxidant capacity. Principal component analysis identified the optimal harvest windows: flowers achieved the highest integrated score at the full blooming stage, whereas leaves scored highest at the early fruiting stage. These periods also corresponded with greater fresh biomass, supporting favorable economic returns. Accordingly, we recommend the full blooming stage as the optimal harvest time for flowers and the early fruiting stage for leaves and stems. Future research should focus on elucidating how environmental factors regulate the accumulation of bioactive compounds, which will further refine cultivation and harvest strategies to enhance the quality of this medicinal herb.

## 1. Introduction

*Penthorum chinense* Pursh, which belongs to the genus *Penthorum* within the family Penthoraceae [1], was first documented in Jiuhuang Bencao (Herbal Relief of Famines), a Ming Dynasty herbal compendium. The geographical distribution of *P. chinense* is primarily concentrated in East Asia, encompassing regions such as eastern Russia, Mongolia, China, Japan, Korea, and Laos [2]. As a traditional folk medicine, it has a long history of medicinal use, with its aboveground parts (stems and leaves) being commonly used in the form of water decoction or as a fresh poultice. Among the Miao ethnic group, this plant is revered as an “immortal herb” given its remarkable hepatoprotective effects, such as liver edema, infectious hepatitis, and liver injuries [2,3,4]. In contemporary commercial practice, the stems, leaves, and flowers are harvested and sold separately, with leaves and flowers commanding substantially higher market prices due to their richer bioactive component profiles. Phytochemical investigations have identified over 100 compounds from *P. chinense*, including polyphenols (e.g., gallic acid, ellagic acid), flavonoids (e.g., pinocembrin, quercetin), and lignans [2]. These constituents have been reported to exhibit hepatoprotective, antioxidant, anti-inflammatory, and anti-fibrotic activities [2].

As modern research has advanced, scholars have progressively elucidated the primary chemical constituents of *P. chinense* and their pharmacological mechanisms of action. Studies have revealed that *P. chinense* primarily contains bioactive compounds such as polyphenols, flavonoids, and coumarins [4]. *P. chinense* was shown to protect the liver from hepatic ischemia–reperfusion injury by inhibiting the HMGB1/TLR4 signaling pathway [5], ameliorate alcoholic liver injury by activating the Ras/Raf/MEK/ERK signaling pathway [6], and exert other pharmacological activity that includes hepatoprotective and anti-inflammatory effects [7,8,9]. In recent years, *P. chinense* has gained increasing attention as a functional food ingredient, driven by growing consumer interest in plant-based products for liver health management. Commercially, it is available in various forms, including herbal teas, dietary supplements, and functional beverages. Notably, *P. chinense* was officially approved as a novel food ingredient in China’s National List in 2020. This significant milestone expanded its application scope and provided new opportunities for further development as well as application of this traditional medicinal plant resource to promote human health.

Selecting the optimal harvest time is a decisive factor in the production of Chinese medicinal materials, directly impacting drug quality, therapeutic efficacy, and resource sustainability. The accumulation of bioactive constituents, which underpins medicinal value, is dynamically regulated by the plant’s phenological stage—from germination and vegetative growth to flowering and fruiting [10]. This dynamic is further modulated by morphological traits (e.g., color, aroma, and size) [11,12,13], phytochemical profiles [14], and environmental conditions [15]. Recent studies emphasize that the biosynthesis and localization of key secondary metabolites—such as flavonoids, terpenoids, and alkaloids—follow distinct spatiotemporal patterns within specific tissues and developmental windows [16,17]. Therefore, a science-based harvest strategy must integrate multiple indicators: phenotypic markers that reflect metabolic shifts, quantitative tracking of bioactive compounds across growth stages, and environmental adaptations [18]. Wang et al. [19] indicated that, considering both quality and yield, it is more reasonable to harvest *B. striata* in late September. Advancing such an integrated approach is essential for standardizing harvest protocols and ensuring the consistent quality of herbal medicines.

*Penthorum chinense* is cultivated on a large scale in Gulin County, Sichuan Province, with a relatively long maturation period spanning from June to September. Although the “Sichuan Standards for Chinese Medicinal Materials” stipulates summer–autumn harvesting for this species, systematic studies employing multiple indicators to determine the precise optimal harvest time have been lacking, leading to widespread inappropriate harvesting practices. In commercial practice, the stems, leaves, and flowers of *P. chinense* are sold separately, with substantially different market prices. Given the growing market demand for *P. chinense*-based products and the absence of standardized harvest protocols, establishing a scientifically validated time-segmented, organ-specific harvest strategy is urgently needed to balance economic value with practical field management. Alcohol dehydrogenase (ADH), a key enzyme involved in alcohol metabolism, serves as a sensitive indicator of liver function and oxidative stress status. In view of the traditional hepatoprotective use of *P. chinense*, ADH activity was selected as a physiologically relevant parameter to evaluate the bioactivity of plant materials harvested at different developmental stages. Accordingly, the purpose of this study is to establish a time-resolved harvesting standard for *P. chinense* by integrating growth phenology, phytochemical accumulation dynamics in stems, leaves, and flowers, and multi-activity (antioxidant and ADH) profiling across six developmental stages. By identifying the optimal harvest window for each plant part and proposing a sequential harvest schedule that aligns with natural phenological progression, this study provides a practical scientific basis for maximizing both economic returns and medicinal quality. Determining organ-specific suitable harvesting periods holds significant practical importance for standardizing cultivation management, ensuring the stability of medicinal material quality, and enhancing the value of related products.

## 2. Materials and Methods

### 2.1. Sample Preparation

*P. chinense* samples were collected from Gulin County, Sichuan Province, on six dates 2024, June 27, July 5, July 20, August 4, August 18, and September 2, covering the major phenological stages from early vegetative growth (late June) through flowering peak (mid- to late July) to early fruiting (August) and late maturation (early September). At each time point, approximately 2 kg of plant material was collected. During the collection period, the average daily temperature ranged from 19 to 37 °C, and the total precipitation was 158.9 mm. The flowers, leaves, and stems were freeze-dried, crushed, and passed through a 60-mesh sieve. The experimental samples are shown in Figure S1.

### 2.2. Chemicals and Reagents

Rutin, Folin–Ciocalteu reagent, phosphomolybdic acid, vanillin (analytically pure), Na_2_CO_3_, Al(NO_3_)_3_, formic acid, NaNO_2_, HCl, alcohol dehydrogenase (265 units/mg), were purchased from Chengdu Kelong Chemical Reagent Factory (Chengdu, China). 2,2′-Azino-bis (3-ethylbenzothiazoline-6-sulfonic acid, ABTS) diammonium salt and standard compounds, including (+)-catechin, (−)-epicatechin, afzelin, astragaline, kaempferol, quercitrin, isoquercitrin, pinocembrin, pinocembrin 7-*O*-glucoside, pinocembrin-7-O-[3″-O-galloyl-4″,6″-(S)-hexahydroxydiphenoyl]-β-D-glucose (PGHG), thonningianin A, gallic acid, and 1,2,3,6-tetragalloylglucose were purchased from Shanghai Yuanye Bio-Technology Co., Ltd. (Shanghai, China). 2,2-Diphenyl-1-picrylhydrazyl (DPPH) was purchased from Shanghai Macklin Biochemical Technology Co., Ltd. (Shanghai, China). Gallotannin 1, phyllanemblinin F, strictinin, and penchinensin A were isolated and identified in our laboratory.

### 2.3. Sample Extraction

Briefly, 1 g of powdered flowers, leaves, and stems of *P. chinense* was accurately homogenized with 8 mL of 80% ethanol and sonicated for 30 min at 40 °C. The sample was then centrifuged at 6654 *g* for 15 min. The supernatant was collected, and the extract was precipitated again with three extractions. The extraction solvent was collected each time, and a 25 mL volumetric flask was used to determine the volume. The samples were stored at −20 °C until later use.

### 2.4. Determination of Active Ingredients

#### 2.4.1. Determination of the Total Phenolic Content

The Folin–Ciocalteu method was used to determine the total phenolic content [20]. Folin–Ciocalteu reagent (20 μL)was added to 20 μL of extracts and left to stand for 5 min. Then, 160 μL of 5% Na_2_CO_3_ was added and reacted for 60 min at room temperature. The absorbance of the mixture was determined at 765 nm by Synergy-HTX Multi-Mode Microplate Reader (BioTek Instruments, Inc., Shoreline, WA, USA). The concentration of gallic acid was plotted on the x-axis, and the absorbance was plotted on the y-axis to create a standard curve with the equation *y* = 0.0046*x* + 0.0034 (*R*^2^ = 0.9914). The total phenolic content mg gallic acid equivalent (GAE)/g Dry Weight (DW) was calculated using the regression equation.

#### 2.4.2. Determination of the Total Flavonoid Content

The NaNO_2_-aluminum(NO)_3_)_3_-NaOH colorimetric method was used to determine the TFC [21]. An amount of 5% NaNO_2_ (15 μL) was mixed with 20 μL of extracts and reacted for 6 min. Then, 10 μL of 10% Al(NO_3_)_3_ was added and reacted for 5 min. Finally, 30 μL of 1 M NaOH was added, and the sample was determined at 510 nm by Synergy-HTX Multi-Mode Microplate Reader. The rutin concentration was plotted on the abscissa and the absorbance on the ordinate to create the standard curve, *y* = 0.4108*x* + 0.0086 (*R*^2^ = 0.9991). From this, the total flavonoids (mg rutin equivalent (RE)/g DW) were calculated using a linear equation.

#### 2.4.3. Determination of the Total Proanthocyanidins Content

The total proanthocyanidins content determination was based on Zhou’s method [22] with slight modifications. Briefly, 1.5 mL of concentrated hydrochloric acid was added to 1 mL of the sample and 3 mL of a 40 g/L vanillin-methanol solution and mixed well. The solution was reacted in the dark at 45 °C for 30 min. After shaking evenly, the optical density (OD) value at 500 nm was determined by Synergy-HTX Multi-Mode Microplate Reader. Using the catechin concentration as the abscissa and absorbance as the ordinate, a standard curve was plotted using *y* = 2.4895*x* + 0.0089 (*R*^2^ = 0.9994). The total proanthocyanidins content (mg catechin equivalent (CE)/g DW) was calculated from the linear equation.

#### 2.4.4. Determination of the Total Tannin Content

To determine the TTC, Missio’s method [23] was used with slight modifications. Briefly, 0.5 mL of the sample was placed into a 25 mL volumetric flask, and 0.85 mL of 70% ethanol and 0.05 mL of a 60 mg/mL metaphosphoric acid solution were added, followed by 12.5 mL of water and 1.25 mL of Folin–Denis reagent. Subsequently, 5 mL of a 1 mol/L sodium carbonate solution was added, and the flask was shaken vigorously. The solution was diluted with water, mixed thoroughly, and incubated in a 39 °C environment for 1.5 h. A standard curve was plotted using the tannic acid content of the standard solution as the abscissa, with the equation *y* = 0.0702*x* + 0.0319 (*R*^2^ = 0.9947). The total tannin content (mg tannic acid equivalent (TAE)/g DW) was calculated based on this linear equation.

#### 2.4.5. Determination of Monomer Phenolic Content

The phenolic content was determined based on Xiang et al. [20] using an Agilent LC-1290 HPLC system (Agilent Corporation, Santa Clara, CA, USA) with an Infinity Lab Poroshell 120 PFP column (4.6 × 100 mm, 2.7 µm particle size, Agilent Corporation). The parameters were detection wavelength, 280 and 320 nm; column temperature, 35 °C; flow rate, 0.8 mL/min; and injection volume, 2 μL. The mobile phase was composed of 0.1% formic acid in water (A) and acetonitrile (B). The gradient elution program was as follows: from 0 to 10 min, B was increased from 5% to 10%; from 10 to 15 min, B was increased from 10% to 20%; from 15 to 20 min, B was increased from 20% to 35%; from 20 to 30 min, B was increased from 35% to 75%; and from 30 to 32 min, B was increased from 75% to 95%.

### 2.5. Determination of Antioxidant Activity

#### 2.5.1. DPPH Free Radical Scavenging Ability

A previously reported DPPH free radical scavenging ability was used with slight modifications [24]. Samples (100 μL) were thoroughly mixed with 100 μL DPPH solution and left to stand in the dark for 30 min. The optical density (OD) value was then measured at 517 nm by Synergy-HTX Multi-Mode Microplate Reader. Vitamin E (VE) was used as the positive control. The standard curve was *y* = 0.0021*x* + 0.0019 (*R*^2^ = 0.9932). The results were expressed as mg Trolox Equivalents (TE)/g DW.

#### 2.5.2. ABTS^+^ Free Radical Scavenging Ability

A previously reported method for determining the ABTS^+^ free radical scavenging ability was used with slight modifications [25]. Briefly, 40 μL of sample and 160 μL of ABTS solution were thoroughly mixed at room temperature and photoresisted for 5 min. The OD was measured at 734 nm by Synergy-HTX Multi-Mode Microplate Reader. VE was used as the positive control. The standard curve was *y* = 0.0022*x* − 0.0442 (*R*^2^ = 0.9909). The results were expressed as mg TE/g DW.

#### 2.5.3. Ferric Reducing Antioxidant Power (FRAP) Assay

Ferric reducing power was determined using the method reported by Zhu et al. [26] with slight modifications. The sample (30 μL) and 265 μL of FRAP working solution were combined. The reaction proceeded for 30 min at 37 °C, and the OD value was measured at 593 nm by Synergy-HTX Multi-Mode Microplate Reader. VE was used as the positive control. The standard curve was *y* = 0.0129*x* − 0.0133 (*R*^2^ = 0.9989). The results were expressed as mg TE/g DW.

#### 2.5.4. Antioxidant Capacity Index

To comprehensively evaluate the antioxidant capacity of the samples, the scavenging rates of DPPH and ABTS^+^ free radicals were measured, and an iron ion reducing power (FRAP) experiment was conducted. An antioxidant potency composite (APC) was used to assess the overall antioxidant efficacy of the samples [27]. A quantitative evaluation was performed, and the antioxidant capacity of each sample was compared to the APC index, with a higher APC index indicating a greater comprehensive antioxidant activity. The calculation was as follows:

(1) APC index (%) = *A*_1_/*A*_Max_ × 100%

(2) Mean APC index (%) = (*APC*_1_ + *APC*_2_ + …*APC*_n_)/n × 100%

where *A*_1_ is the value determined by this method, *A*_Max_ is the maximum value determined by this method, *APC*_1_ is the APC index value from method 1, *APC*_2_ is APC index value from method 2, and *APC*_n_ is APC index value from method n.

### 2.6. Alcohol Dehydrogenase (ADH) Activity

The Valle and Hoch method [28] was used with modifications to determine ADH activity. Briefly, 1.5 mL of sodium diphosphate buffer (32 mM, pH 8.8), 1 mL of NAD coenzyme solution (27 mM), 0.5 mL of ethanol (11.5%), and 100 μL of a sample were sequentially added to a tube and incubated at 37 °C for 5 min. Then, 100 μL of an enzyme solution was added and the mixture was shaken well. A spectrophotometer was immediately used to measure the absorbance at 340 nm every 10 s for 5 min until the absorbance per minute reached a stable value. For the blank, 100 μL of distilled water was used in place of the sample. The calculation was as follows:

(3) E = (∆A340 × V)/(E_W_ × 6.2)

(4) R = (E1 − E0)/E0

where E is the enzyme activity, U/mL; ΔA340 is the increase in absorbance per 1 min at a wavelength of 340 nm; Ew is the activity of the added ADH enzyme, U/mL; 6.2 is the molar extinction coefficient of NADH at 340 nm; V is the total volume of the reaction solution, mL; R is the activation rate of the alcohol-degrading enzyme by the sample, %; E1 is the enzyme activity of the sample solution, U/mL; and E0 is the enzyme activity of the blank, U/mL.

### 2.7. Statistical Analysis

All experiments were performed with three biological replicates unless otherwise specified. The data obtained from phytochemical analyses and antioxidant activity assays across the six harvesting periods were subjected to one-way analysis of variance (ANOVA) followed by Duncan’s multiple range test using SPSS 17.0 software (version 17.0, SPSS Inc., Chicago, IL, USA), with a significance level set at *p* < 0.05. Principal component analysis (PCA) was further conducted to integrate multiple growth, physiological, and biochemical indicators, enabling the comprehensive evaluation and ranking of different harvesting periods and plant tissues.

## 3. Results

### 3.1. Physiological Properties

The growth cycle of *P. chinense* comprised four stages: vegetative growth (June 27–July 5), reproductive growth (July 20), fruiting (August 4–18), and senescence (September 2) (Table 1). Stem length increased from 65.61 cm on June 27 to a peak of 111.17 cm on August 18, with a transient decrease on August 4 during high-temperature stress (>35 °C). Fresh weight peaked at 126.81 g on July 20, then gradually declined. Dry matter ratio was lowest on August 18 (23.54%) and highest on September 2 and July 5 (33.24%, 32.69%).

### 3.2. Total Phenolic, Flavonoid, Proanthocyanidin, and Tannin Content

**Figure 1:**
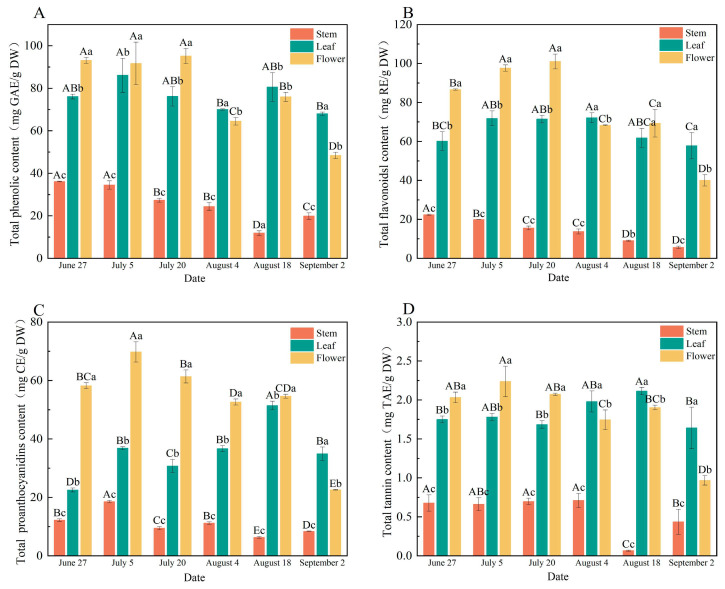
The total polyphenols (**A**), total flavonoids (**B**), total proanthocyanidins (**C**) and total tannins (**D**) of different parts of *P. chinense* at different harvest time. Different capital letters indicate significant differences in the same part among different harvest periods, while different lowercase letters indicate significant differences in the same harvest period among different parts.

The accumulation of bioactive compounds in *P. chinense* exhibited significant temporal dynamics and tissue-specific variation throughout the growth cycle (Figure 1). Overall, the content of total phenolics, flavonoids, proanthocyanidins, and tannins followed a complex “increase–decrease–increase–decrease” pattern across maturation.

Total phenolic and flavonoid contents showed rapid accumulation from June to July, both peaking simultaneously on July 20 at 95.16 mg GAE/g DW and 101.02 mg RE/g DW, respectively. In contrast, total proanthocyanidins and tannins reached their maxima earlier on July 5, with contents of 69.74 mg CE/g DW and 2.24 mg TAE/g DW. A notable transient re-accumulation of all compounds occurred around August 18, coinciding with the transition from flowering to initial fruiting, followed by a final decline by late August. By September 2, proanthocyanidin and tannin contents had decreased significantly to 22.55 mg CE/g DW and 0.97 mg TAE/g DW, representing reductions of 67.7% and 56.7%, respectively.

Bioactive compound accumulation followed a consistent tissue-specific order: flower > leaf > stem (Figure 1). Flowers contained the highest levels of all measured metabolites, with all four components peaking synchronously in mid-to-late July. Leaves exhibited distinct accumulation patterns: total phenolics peaked earliest on July 5 (86.13 mg/g), total flavonoids remained stable from July to early August (average 71.43 mg/g, *p* > 0.05) before declining, while proanthocyanidins and tannins reached their highest levels on August 18. Stems showed the lowest accumulation, with maximum total phenolic and tannin contents of only 36.14 mg/g and 0.68 mg/g, respectively, which decreased markedly by 67.2% and 89.7% as growth progressed. Stem tannin content remained relatively stable until late August, suggesting distinct regulatory mechanisms.

### 3.3. Quantification of Phenolic Compounds

A systematic time-course study was conducted to analyze the dynamic changes of 18 monomeric phenolic compounds in *P. chinense* across different harvesting dates (Table 2). The results revealed significant seasonal variations in the accumulation of active constituents, with distinct patterns observed among flowers, leaves, and stems.

The 18 phenolic monomers in *P. chinense* showed distinct tissue-specific patterns. Leaves contained higher penthorumin A, PGHG, afzelin, and catechin. Flowers were enriched in epicatechin, isoquercitrin, kaempferol-3-*O*-rutinoside, kaempferol, and quercetin. Stems featured quercetin, PGHG, and pinostrobin-7-*O*-glucoside. Rutin was detected only in flowers and leaves except on September 2. On July 5, hepatoprotective compounds (kaempferol, pinostrobin, gallic acid, quercetin) peaked across all tissues. Thereafter, gallic acid, catechin, kaempferol, quercetin, and afzelin in flowers declined continuously, while kaempferol-3-*O*-rutinoside, astragalin, pinostrobin-7-*O*-glucoside, and rutin showed “increase–decrease–re-increase” patterns. Thonningianin A in flowers increased to 61.98 mg/g DW on August 18, then declined sharply, while concurrently decreasing in leaves and stems. This coincided with leaf yellowing and stem darkening (Figure S1), suggesting translocation to reproductive organs during senescence and fruit maturation.

### 3.4. Antioxidant Activity

**Figure 2:**
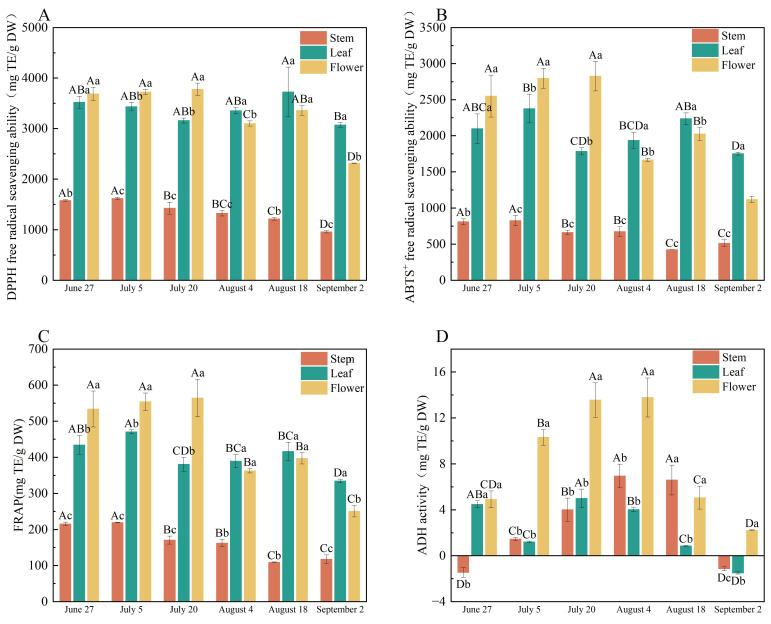
DPPH (**A**) and ABTS^+^ (**B**) free radical scavenging ability; FRAP (**C**) and ADH (**D**) activity of different parts of *P. chinense* at different harvest time. Different capital letters indicate significant differences in the same part among different harvest periods, while different lowercase letters indicate significant differences in the same harvest period among different parts.

The antioxidant activity of *P. chinense* was evaluated using DPPH, ABTS^+^, and FRAP assays, revealing significant spatiotemporal variation across tissues and harvest dates (*p* < 0.05) (Figure 2). Flowers exhibited the highest DPPH scavenging capacity from late June to mid-July, followed by a marked decline. Leaf activity peaked in mid-August, while stems showed continuous decline throughout sampling. Flower samples from July 20 and leaf samples from August 18 demonstrated the highest activity. Flower scavenging capacity increased from June, peaking on July 20, then declined sharply by 60.40% by September 2. Leaf activity peaked on July 5 and August 18. Stems showed minimal variation, indicating stable low-level antioxidant content. Flower reducing power peaked on July 20, significantly exceeding leaves and stems, followed by a sharp decline after early August. Leaf activity remained stable from June to July, with a slight post-August increase. Stems maintained consistently low activity throughout. Comprehensive antioxidant capacity (APC index): Integrated analysis of the three assays (Table S1) showed significant differences among harvest dates (*p* < 0.05). Flower samples from July 20 exhibited the highest APC index, indicating superior comprehensive antioxidant capacity, followed by flowers from July 5 and June 18. Among leaves, samples from July 5 and August 18 showed the highest activity.

### 3.5. ADH Activity

ADH activity in *P. chinense* exhibited distinct temporal patterns across tissues (Figure 2D). Flowers showed a unimodal trend, increasing from June 27 to peak on July 20, then declining thereafter. Leaves followed a similar pattern with maximum activity on July 20. In contrast, stems peaked later on August 4. The rise in ADH activity from June 27 to July 20 coincided with vegetative and reproductive growth stages. Notably, this temporal variation closely paralleled the accumulation dynamics of key secondary metabolites in corresponding tissues.

### 3.6. Principal Component Analysis

**Figure 3:**
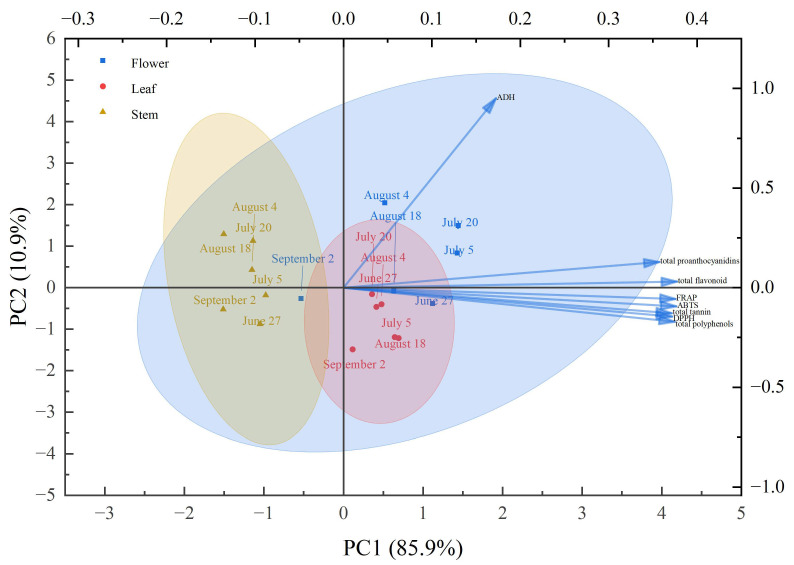
Principal component analysis of active ingredients in *P. chinense* in relation to different harvest periods and plant parts.

PCA was performed on eight phytochemical and antioxidant parameters across harvest periods to determine optimal harvest timing for *P. chinense*. Data suitability was confirmed (Kaiser–Meyer–Olkin (KMO) = 0.847). One principal component (PC1) with eigenvalue > 1 was extracted, explaining 85.92% of total variance (Figure 3). Loading analysis showed that total phenolics, flavonoids, proanthocyanidins, tannins, and antioxidant indicators were strongly associated with PC1, indicating that PC1 represents the integrated biochemical quality profile. ADH showed moderate contributions to both components. The score plot revealed distinct chemotypic patterns. Flower samples were widely dispersed across quadrants, indicating strong harvest time dependence. Leaf and stem samples formed tighter clusters, suggesting less temporal sensitivity. Based on composite scores, flowers peaked on July 20, leaves on August 18, and stems on July 5.

## 4. Discussion

The growth pattern of *P. chinense* reflects developmental programming modulated by environmental factors. Rapid biomass accumulation until July 20 indicates efficient photosynthesis and nitrogen assimilation during vegetative and reproductive growth [29,30]. The transient stem contraction on August 4 represents a reversible turgor-mediated response to high-temperature-induced water stress [31]. Post-reproductive biomass decline corresponds to nutrient reallocation to developing fruits. The inverse relationship between fresh weight and dry matter ratio during fruiting and senescence reflects progressive dehydration and tissue lignification. These physiological changes inform harvest timing: peak fresh weight (July 20) maximizes fresh market yield, while maximum dry matter ratio (September 2) optimizes processing efficiency. Local market data (13.3 t/ha fresh yield; 4:1 fresh-to-dry ratio) support September harvest for dry herb production to maximize economic returns given fresh market prices of 6–8 yuan/kg. This study evaluated harvest timing based on dry weight content of bioactive components. We acknowledge that total yield per plant is another important consideration for producers, and future studies should integrate biomass dynamics to establish more comprehensive harvest guidelines.

The accumulation patterns of bioactive compounds in *P. chinense* reflect complex interactions between developmental programming and environmental regulation, with direct implications for optimal harvest timing. The initial rapid accumulation aligns with active floral development, where secondary metabolites serve protective functions. The synchronous peak of phenolics and flavonoids on July 20 identifies this as the optimal harvest window for these medicinally valuable compounds. The earlier peaks of proanthocyanidins and tannins (July 5) suggest differential regulation within the phenylpropanoid pathway. Post-peak declines, particularly the 67.7% reduction in proanthocyanidins by September 2, likely result from (1) dilution effects from rapid biomass accumulation; (2) nutrient reallocation to developing fruits during reproductive transition [32]; and (3) tissue senescence. The transient re-accumulation around August 18 represents a brief metabolic adjustment before final decline. Decreasing tannin content, associated with reduced astringency [33], reflects broader shifts in secondary metabolism during maturation, confirming the critical link between harvest timing and therapeutic efficacy [34,35]. Light and temperature modulate the phenylpropanoid pathway by influencing key enzymes (Phenylalanine Ammonia-Lyase (PAL), Cinnamate 4-Hydroxylase (C4H), 4-Coumarate-CoA Ligase (4CL)) [36,37]. Light specifically regulates accumulation through direct upregulation of biosynthetic genes (*Malus domestica* Anthocyanidin Synthase **(**MdANS), *Malus domestica* UDP-Flavonoid Glucosyltransferase **(**MdUFGT)) and Myeloblastosis family transcription factor **(**MYB) transcription factors [38]; ethylene-mediated signaling [39]; and promotion of proanthocyanidin conversion to anthocyanins via (Chalcone Synthase) CHS, Dihydroflavonol 4-Reductase **(**DFR), and Anthocyanidin Synthase **(**ANS) upregulation [40,41]. Light also interacts with abscisic acid and jasmonate signaling [42]. These networks likely contributed to observed fluctuations coinciding with seasonal environmental changes.

Flowers invest heavily in phenolics for reproduction-related protection and signaling, with synchronized peaking indicating coordinated pathway regulation. Leaves exhibited distinct patterns: early phenolic peak (July 5) for photoprotection; sustained flavonoids through early August; and late proanthocyanidin/tannin peak (August 18) suggesting stress response or remobilization. Stems showed minimal accumulation, with stable tannins until late August indicating constitutive defense rather than developmental regulation. These patterns confirm flowers as the primary accumulation site [43] and support harvesting flowering tops in mid-to-late July to maximize phenolic and flavonoid yields for medicinal applications.

The distinct tissue-specific distribution of phenolic monomers reflects functional specialization: leaves accumulate defensive compounds, flowers enrich reproductive-associated metabolites, and stems maintain constitutive defenses [44]. These patterns support the traditional Chinese medicine principle of using specific plant parts for targeted therapeutic applications. The July 5 peak of hepatoprotective compounds identifies this as the optimal harvest window for maximizing these metabolites [45,46]. The contrasting temporal patterns among compounds suggest differential regulation within the phenylpropanoid pathway. Notably, thonningianin A accumulation in flowers concurrent with its decline in leaves and stems indicates active translocation from vegetative to reproductive tissues during reproductive transition [47], confirmed by coinciding phenotypic changes. The subsequent sharp decline and redistribution to developing fruits represents final resource allocation before senescence. These findings guide harvest optimization: early July for hepatoprotective compounds, mid-August for thonningianin A from flowers.

The significant spatiotemporal variation in antioxidant activity across *P. chinense* tissues closely mirrors the accumulation patterns of phenolic compounds [48], confirming that phenolic-rich tissues exhibit enhanced radical scavenging and reducing power via ortho-phenolic hydroxyl-mediated oxidation mechanisms [49,50,51]. The temporal decline in flower activity after July 20 reflects senescence-associated compound degradation and metabolic redistribution during reproductive transition, consistent with observations in Adlay [52] and *Peucedanum japonicum* [53]. The 60.40% reduction in ABTS^+^ activity by September 2 underscores the rapid loss of hydrophilic and lipophilic antioxidants during late maturation. While previous studies noted growth stage-dependent variation in tissue-specific antioxidant activity [54,55,56], our integrated APC index provides a comprehensive ranking. The superior APC of July 20 flowers, followed by July 5 flowers and August 18 leaves, establishes clear harvest priorities: flowering tops in mid-to-late July for maximum antioxidant yield, and leaves in early July or mid-August for specific applications. These findings provide a scientific basis for optimizing harvest strategies to meet different product requirements.

The unimodal ADH activity pattern in flowers and leaves, peaking on July 20, aligns with hormone-mediated metabolic activation during reproductive growth, likely involving auxin and cytokinin signaling [57]. The later stem peak (August 4) suggests tissue-specific regulatory mechanisms. The parallel temporal dynamics between ADH activity and secondary metabolite accumulation indicates potential metabolic coordination, supporting ADH activity as a reliable physicochemical indicator for quality assessment. As ADH catalyzes ethanol-to-acetaldehyde conversion, enhanced activity correlates with improved ethanol clearance [58]. This validates the traditional use of *P. chinense* for liver protection and establishes July 20 as the optimal harvest window for maximizing therapeutic potential related to alcohol detoxification.

PCA effectively distinguished *P. chinense* samples by tissue type and harvest time, explaining 85.92% of variance in a single component representing integrated biochemical quality. The strong loading of phenolic compounds and antioxidant parameters on PC1 confirms their collective contribution to overall phytochemical profile, consistent with previous findings [59]. The wider dispersion of flower samples across quadrants indicates greater temporal sensitivity in reproductive tissues, where secondary metabolism shifts dramatically during flowering and fruit set. In contrast, tighter clustering of leaves and stems suggests more stable biochemical profiles in vegetative tissues, reflecting their constitutive rather than developmentally regulated metabolic roles. These tissue-specific temporal dynamics provide a rational basis for harvest optimization: July 20 for flowers, aligning with peak phenolic accumulation (Section 3.2) and antioxidant activity (Section 3.4); August 18 for leaves, coinciding with late-season metabolite peaks; and July 5 for stems. This multi-tissue harvest strategy maximizes overall therapeutic potential and supports sustainable utilization of *P. chinense* resources.

## 5. Conclusions

Currently, harvesting of *P. chinense* lacks unified standards. To our knowledge, this study is the first to evaluate growth, physiological, and biochemical indicators across six harvesting periods. The content of bioactive compounds exhibited temporal and spatial variation. Based on these findings, we provide the following recommendations: growers should harvest flowers at full bloom (around July 20) and leaves/stems during early fruiting (around August 18) to optimize bioactive content and yield; processors are advised to adopt stage-specific and tissue-separated handling to preserve quality. During the sampling period (June 27 to September 2, 2024), temperatures ranged from 19 °C to 37 °C with total precipitation of approximately 159 mm. As these timeframes may require local calibration under different climatic conditions, future research should investigate how environmental factors influence compound synthesis, validate the economic feasibility of the proposed strategies, and optimize post-harvest processing methods.

## Figures and Tables

**Table 1:** The average length, fresh weight and dry matter percentage of *P. chinense.*

Harvest Time	Average Length (cm)	Fresh Weight (g)	Dry Matter Percentage (%)
June 27	65.61 ± 6.14 ^ab^	49.16 ± 5.90 ^b^	25.07 ± 1.27 ^cd^
July 5	71.29 ± 6.64 ^ab^	53.46 ± 7.15 ^b^	32.69 ± 1.37 ^a^
July 20	98.15 ± 9.32 ^ab^	126.81 ± 11.44 ^a^	29.12 ± 1.31 ^b^
August 4	77.38 ± 10.10 ^ab^	67.05 ± 5.07 ^b^	28.10 ± 0.83 ^bc^
August 18	111.17 ± 10.77 ^a^	94.48 ± 7.25 ^a^	23.54 ± 0.91 ^d^
September 2	95.36 ± 13.13 ^ab^	50.48 ± 6.38 ^b^	33.24 ± 1.65 ^a^

Note: Different lowercase letters represent significant differences in harvest periods.

**Table 2:** The content of phenolic compounds in different parts and different harvest periods of *P. chinense* (mg/DW g).

Date	Part	Gallic Acid	Catechin	Epicatechin	Isoquercitrin	Kaempferol-3-Rutinoside	Astragalin	Pinocembrin 7-O-beta-D-glucoside	Pinocembrin-7-o-(3″-galloyl-4″,6″-(s)-hexahydroxydiphenoyl)-β-d-glucose	Pinocembrin	Thonningianin A	Rutin	Afzelin	Quercetin	Kaempferol	Gallotannin	Phyllanemblinin F	Strictinin	Penchinensin A
June 27	Stem	0.51 ± 0.00 ^Ea^	2.01 ± 0.01 ^Ha^	0.31 ± 0.01 ^Aa^	0.12 ± 0.00 ^Aa^	0.22 ± 0.02 ^Ca^	0.39 ± 0.07 ^Ea^	8.56 ± 0.05 ^Jb^	11.91 ± 0.09 ^Kb^	0.09 ± 0.00 ^Ga^	4.84 ± 0.00 ^Ia^	/	0.23 ± 0.01 ^Ca^	0.81 ± 0.01 ^Ic^	0.34 ± 0.00 ^DEc^	3.87 ± 0.08 ^Cb^	6.57 ± 0.05 ^Aa^	2.23 ± 0.02 ^Ca^	4.52 ± 0.04 ^B^
	Leaves	0.64 ± 0.00 ^Ac^	4.41 ± 0.24 ^Cc^	0.60 ± 0.03 ^Ab^	0.82 ± 0.04 ^Ab^	0.43 ± 0.08 ^Aa^	1.40 ± 0.23 ^ABb^	2.92 ± 0.03 ^BCa^	6.93 ± 0.58 ^Da^	0.37 ± 0.16 ^Ab^	100.71 ± 3.58 ^Ec^	0.08 ± 0.00 ^A^	1.59 ± 0.000 ^ABc^	0.10 ± 0.00 ^a^	0.11 ± 0.00 ^Aa^	9.56 ± 0.02 ^Aa^	5.12 ± 0.02 ^Bd^	7.99 ± 0.02 ^Aa^	4.51 ± 0.03 ^Bd^
	Flower	0.61 ± 0.01 ^BCb^	3.64 ± 0.07 ^Fb^	0.81 ± 0.04 ^CDc^	1.27 ± 0.13 ^BCc^	1.31 ± 0.10 ^DEb^	1.63 ± 0.24 ^Eb^	7.34 ± 0.91 ^Gb^	19.95 ± 0.30 ^Hc^	0.28 ± 0.01 ^ABab^	44.31 ± 0.11 ^Ib^	0.05 ± 0.00 ^A^	0.80 ± 0.04 ^CDb^	0.72 ± 0.02 ^Db^	0.45 ± 0.04 ^ABCb^	6.70 ± 0.05 ^Ba^	4.72 ± 0.09 ^Cbc^	4.07 ± 0.08 ^Ba^	5.71 ± 0.12 ^Ab^
July 5	Stem	0.51 ± 0.00 ^Da^	1.78 ± 0.00 ^Ea^	0.39 ± 0.00 ^Aa^	0.26 ± 0.00 ^BCa^	0.27 ± 0.01 ^BCa^	0.43 ± 0.03 ^CDa^	8.87 ± 0.01 ^Hb^	10.43 ± 0.35 ^Jb^	0.05 ± 0.01 ^Aa^	4.91 ± 0.04 ^Ga^	/	0.09 ± 0.01 ^Aa^	0.42 ± 0.00 ^CDb^	0.21 ± 0.00 ^ABb^	5.30 ± 0.04 ^Ba^	5.99 ± 0.06 ^Abc^	2.05 ± 0.02 ^Cb^	4.34 ± 0.01 ^C^
	Leaves	0.64 ± 0.01 ^Ab^	5.11 ± 0.17 ^Bc^	0.63 ± 0.03 ^Ab^	0.45 ± 0.03 ^Aa^	0.51 ± 0.04 ^Ab^	1.52 ± 0.10 ^Ab^	3.85 ± 0.05 ^Ba^	7.5 ± 0.55 ^Ca^	0.40 ± 0.07 ^Ab^	108.02 ± 3.59 ^Dc^	0.07 ± 0.00 ^A^	1.19 ± 0.03 ^Ac^	0.11 ± 0.00 ^Aa^	0.12 ± 0.00 ^Aa^	7.96 ± 0.22 ^Ab^	5.26 ± 0.09 ^Bd^	8.33 ± 0.25 ^Aa^	4.59 ± 0.06 ^Bd^
	Flower	0.64 ± 0.00 ^CDb^	4.45 ± 0.03 ^Gb^	0.69 ± 0.02 ^CDb^	1.22 ± 0.21 ^Eb^	1.35 ± 0.00 ^Ec^	1.68 ± 0.18 ^Fb^	9.00 ± 0.01 ^Hc^	15.28 ± 0.19 ^Ic^	0.38 ± 0.02 ^Bb^	41.22 ± 0.44 ^Jb^	/	0.80 ± 0.00 ^Db^	0.52 ± 0.00 ^BCc^	0.30 ± 0.02 ^Bc^	4.97 ± 0.03 ^Bc^	4.59 ± 0.02 ^Cbc^	3.28 ± 0.01 ^Bb^	8.53 ± 0.07 ^Aa^
July 20	Stem	0.55 ± 0.01 ^Ba^	1.02 ± 0.01 ^Ca^	/	0.02 ± 0.04 ^Aa^	0.15 ± 0.01 ^ABa^	0.22 ± 0.03 ^ABa^	6.78 ± 0.12 ^Eb^	9.25 ± 0.74 ^Fa^	/	3.87 ± 0.20 ^Da^	/	/	0.22 ± 0.01 ^ABc^	0.18 ± 0.00 ^ABb^	0.76 ± 0.01 ^Ad^	5.47 ± 0.03 ^Cc^	1.68 ± 0.01 ^Cd^	4.56 ± 0.02 ^B^
	Leaves	0.64 ± 0.00 ^ABc^	3.31 ± 0.09 ^Cc^	1.39 ± 0.10 ^Ba^	0.35 ± 0.02 ^ABb^	0.38 ± 0.03 ^ABb^	1.07 ± 0.06 ^ABb^	2.61 ± 0.04 ^Ca^	7.58 ± 0.35 ^Da^	/	97.44 ± 2.29 ^Fc^	0.04 ± 0.00 ^A^	1.18 ± 0.02 ^ABb^	0.05 ± 0.00 ^Aa^	0.14 ± 0.00 ^Aa^	2.38 ± 0.22 ^Bd^	15.41 ± 0.43 ^Aa^	5.56 ± 0.12 ^Ab^	7.75 ± 0.12 ^Aa^
	Flower	0.60 ± 0.01 ^ABCb^	1.44 ± 0.04 ^DEb^	2.30 ± 0.17 ^Fb^	1.47 ± 0.05 ^DEc^	1.12 ± 0.05 ^CDc^	0.95 ± 0.03 ^Cb^	2.22 ± 0.05 ^EFa^	16.94 ± 0.65 ^Gb^	/	45.94 ± 1.50 ^Hb^	0.03 ± 0.00 ^A^	0.59 ± 0.02 ^ABCa^	0.21 ± 0.01 ^ABb^	0.14 ± 0.00 ^Aa^	2.36 ± 0.11 ^Ae^	11.84 ± 0.41 ^Ba^	2.84 ± 0.28 ^Bd^	8.58 ± 1.26 ^Aa^
August 4	Stem	0.67 ± 0.02 ^Bc^	1.61 ± 0.01 ^Cb^	/	0.08 ± 0.00 ^Aa^	0.18 ± 0.01 ^ABa^	0.52 ± 0.01 ^ABa^	8.64 ± 0.50 ^Eb^	13.38 ± 0.86 ^Fb^	0.03 ± 0.02 ^A^	3.35 ± 0.11 ^Da^	/	0.12 ± 0.00 ^Aa^	0.17 ± 0.01 ^AB^	0.19 ± 0.01 ^ABc^	1.45 ± 0.11 ^Cc^	4.34 ± 0.08 ^Ce^	2.06 ± 0.06 ^Cb^	3.98 ± 0.04 ^C^
	Leaves	0.61 ± 0.01 ^BCb^	3.72 ± 0.04 ^Gc^	0.77 ± 0.05 ^Cb^	0.37 ± 0.01 ^Bb^	0.55 ± 0.01 ^BCb^	1.53 ± 0.02 ^Ec^	2.58 ± 0.05 ^Fa^	8.72 ± 0.26 ^Ha^	/	86.23 ± 0.47 ^Ec^	0.04 ± 0.00 ^A^	1.14 ± 0.02 ^Dc^	/	0.10 ± 0.00 ^Aa^	3.36 ± 0.17 ^Ac^	9.35 ± 0.39 ^Abc^	5.12 ± 0.11 ^Ab^	4.95 ± 0.11 ^Ac^
	Flower	0.58 ± 0.01 ^ABa^	1.20 ± 0.02 ^Ba^	0.45 ± 0.01 ^ABa^	1.02 ± 0.01 ^Bc^	0.71 ± 0.09 ^ABc^	0.71 ± 0.01 ^ABb^	2.09 ± 0.05 ^Ca^	22.49 ± 0.46 ^Dc^	0.09 ± 0.01 ^A^	50.22 ± 1.48 ^Fb^	0.10 ± 0.00 ^A^	0.45 ± 0.01 ^ABb^	0.08 ± 0.01 ^A^	0.12 ± 0.00 ^Ab^	2.78 ± 0.04 ^Bd^	5.01 ± 0.06 ^Bbc^	2.58 ± 0.04 ^Bd^	4.58 ± 0.04 ^Bb^
August 18	Stem	0.43 ± 0.01 ^Ca^	0.93 ± 0.03 ^Ba^	/	/	/	0.20 ± 0.03 ^ABa^	3.81 ± 0.07 ^Fb^	4.622 ± 0.04 ^Ga^	/	1.36 ± 0.02 ^Ea^	/	/	0.10 ± 0.00 ^ABb^	0.13 ± 0.00 ^ABb^	1.56 ± 0.28 ^Bc^	5.04 ± 0.05 ^Bd^	1.76 ± 0.02 ^Cc^	4.05 ± 0.06 ^B^
	Leaves	0.56 ± 0.00 ^ABb^	4.52 ± 0.11 ^Cc^	0.60 ± 0.02 ^Aa^	0.38 ± 0.003 ^A^	0.53 ± 0.03 ^A^	1.70 ± 0.04 ^Bc^	3.86 ± 0.08 ^Cb^	9.81 ± 0.20 ^Db^	0.25 ± 0.04 ^A^	95.21 ± 2.06 ^Ec^	0.05 ± 0.00 ^A^	1.05 ± 0.02 ^AB^	0.06 ± 0.00 ^Aa^	0.11 ± 0.00 ^Aa^	2.04 ± 0.18 ^Bd^	7.69 ± 0.08 ^Ac^	6.55 ± 0.13 ^Aab^	4.44 ± 0.03 ^Ad^
	Flower	0.56 ± 0.00 ^ABb^	1.40 ± 0.02 ^Bb^	1.33 ± 0.01 ^Bb^	/	0.89 ± 0.26 ^AB^	0.72 ± 0.00 ^ABb^	2.59 ± 0.01 ^Ca^	25.11 ± 0.91 ^Dc^	0.07 ± 0.01 ^A^	61.98 ± 1.65 ^Eb^	0.11 ± 0.00 ^A^	0.44 ± 0.01 ^AB^	0.18 ± 0.01 ^Ac^	0.15 ± 0.001 ^Ac^	5.31 ± 0.03 ^Ab^	4.35 ± 0.02 ^Cc^	3.24 ± 0.01 ^Bb^	4.51 ± 0.04 ^Ab^
September 2	Stem	0.50 ± 0.00 ^BCa^	0.70 ± 0.02 ^Ca^	/	/	0.12 ± 0.01 ^Aa^	0.26 ± 0.02 ^Aa^	5.88 ± 0.21 ^Ec^	6.63 ± 0.53 ^Fa^	/	1.90 ± 0.18 ^Da^	0.09 ± 0.01 ^Ab^	/	0.14 ± 0.01 ^Ac^	0.16 ± 0.06 ^ABc^	0.54 ± 0.04 ^Bd^	4.34 ± 0.12 ^e^	1.69 ± 0.01 ^Bd^	4.59 ± 0.62 ^B^
	Leaves	0.71 ± 0.01 ^Ec^	2.98 ± 0.01 ^Hb^	0.53 ± 0.01 ^DEa^	0.30 ± 0.00 ^Ca^	0.36 ± 0.03 ^CDc^	1.12 ± 0.03 ^Gb^	3.25 ± 0.06 ^Ib^	7.74 ± 0.13 ^Jb^	0.25 ± 0.01 ^C^	95.28 ± 0.34 ^Kc^	0.03 ± 0.00 ^Aa^	0.90 ± 0.10 ^F^	0.04 ± 0.00 ^Aa^	0.10 ± 0.00 ^ABb^	3.24 ± 0.17 ^Ac^	7.93 ± 0.22 ^Ac^	4.18 ± 2.02 ^c^	6.03 ± 0.06 ^Ab^
	Flower	0.53 ± 0.00 ^Ab^	/	0.51 ± 0.02 ^Aa^	0.54 ± 0.01 ^Ab^	0.20 ± 0.00 ^Ab^	0.32 ± 0.01 ^Aa^	1.51 ± 0.04 ^Aa^	16.08 ± 0.05 ^Bc^	0.17 ± 0.01 ^A^	36.28 ± 5.56 ^Cb^	0.04 ± 0.00 ^Aa^	0.22 ± 0.01 ^A^	0.09 ± 0.00 ^Ab^	0.09 ± 0.00 ^Aa^	0.23 ± 0 ^Cf^	4.33 ± 0.01 ^c^	2.23 ± 0.01 ^Be^	4.52 ± 0.03 ^Bb^

Note: Uppercase letters have significant differences in the same part at different harvest times, and lowercase letters have significant differences in different parts at the same time. “/” indicates that it is not checked out.

## Data Availability

The original contributions presented in this study are included in the article/Supplementary Materials. Further inquiries can be directed to the corresponding authors.

## Supplementary Materials

The following supporting information can be downloaded at: https://www.mdpi.com/article/10.3390/foods15081371/s1, Figure S1: Growth changes of *P. chinense* in different harvest periods; Table S1: Analysis of antioxidant comprehensive index at different harvest dates and different parts of *P. chinense*.
