# Supplementary material for: Changes in Active Components, Antioxidant Activity and Alcohol Dehydrogenase Activity of Penthorum chinense Pursh at Different Harvest Times
Source: Foods. 2026 Apr 15;15(8):1371. doi: 10.3390/foods15081371 (PMC13115275; doi:10.3390/foods15081371)
Supplement: Supplementary file 1 [file foods-15-01371-s001.zip › foods-4210657-supplementary.pdf]

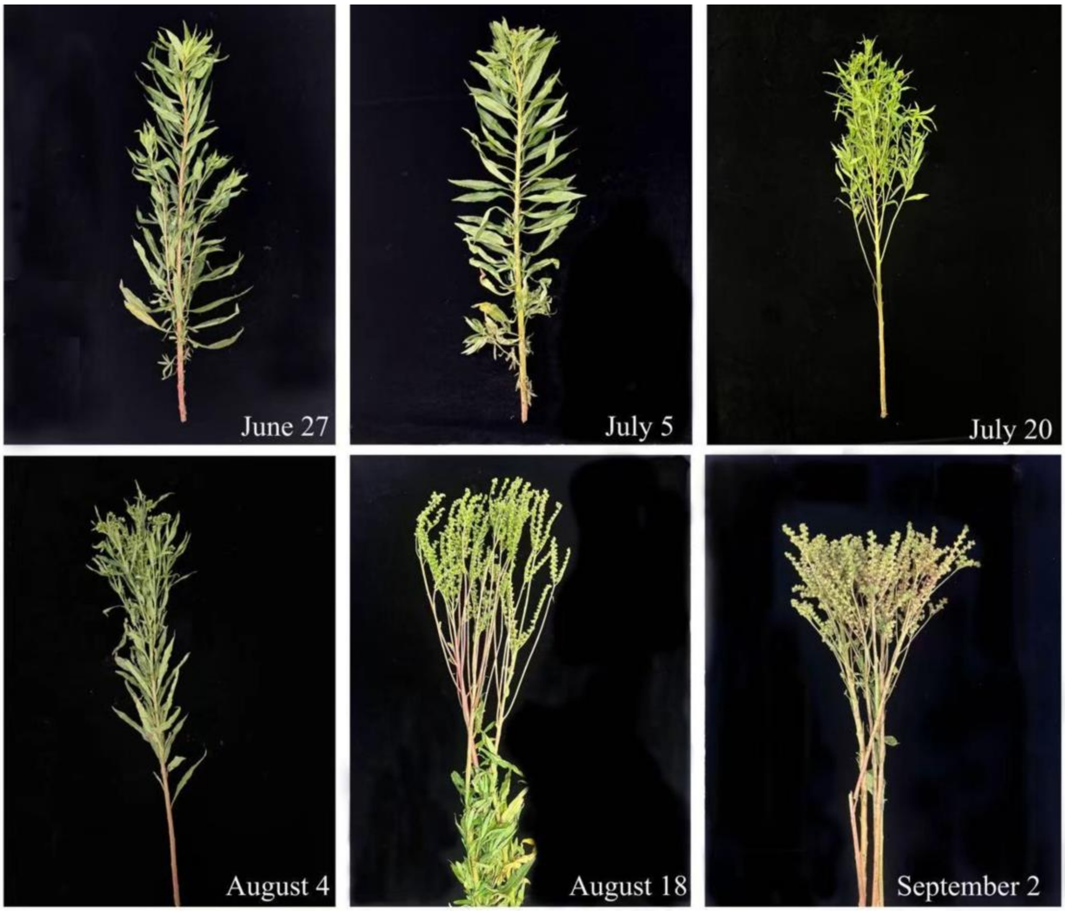

Figure S1 Growth changes of *P. chinense* in different harvest periods

Table S1 Analysis of antioxidant comprehensive index at different harvest dates and different parts of *P. chinense*

Date	Parts	DPPH(mg/g)	ABTS(mg/g)	FRAT(mg/g)	APC	Sort
June 27	Leaves	3519.07	2097.83	434.41	81.48	6
	Stem	1576.72	808.23	215.38	36.18	14
	Flower	3685.55	2549.54	533.60	94.14	3
July 5	Leaves	3433.49	2375.36	470.18	86.12	4
	Stem	1616.44	825.44	219.06	36.95	13
	Flower	3720.09	2796.41	553.81	98.55	2
July 20	Leaves	3156.87	1783.27	380.65	71.40	9
	Stem	1425.02	663.60	170.54	30.48	15
	Flower	3777.11	2825.47	563.95	100.00	1
August 4	Leaves	3353.19	1936.57	389.65	75.47	8
	Stem	1323.04	674.48	162.43	29.23	16
	Flower	3099.23	1664.05	362.65	68.42	10
August 18	Leaves	3724.97	2235.63	416.29	83.85	5
	Stem	1212.26	423.52	108.87	22.13	17
	Flower	3358.82	2024.89	397.03	77.00	7
September 2	Leaves	3069.32	1751.87	334.46	67.52	11
	Stem	959.10	512.82	117.57	21.46	18
	Flower	2311.99	1118.85	250.62	48.42	12
